# The footwear experiences of people with gout: a qualitative study

**DOI:** 10.1186/s13047-019-0349-7

**Published:** 2019-07-12

**Authors:** Mike Frecklington, Anita Williams, Nicola Dalbeth, Peter McNair, Peter Gow, Keith Rome

**Affiliations:** 1 Health and Rehabilitation Research Institute, AUT University, Private Bag 92006, Auckland, 1142 New Zealand; 2 School of Health Science, University of Salford, Salford, UK; 3 The University of Auckland and Auckland District Health Board, Auckland, New Zealand; 4 Counties Manukau District Health Board, Auckland, New Zealand

**Keywords:** Gout, Footwear, Qualitative research

## Abstract

**Background:**

Footwear is an important concern for people with gout, who often describe difficulty finding suitable footwear. Previous studies have identified footwear as a major concern for people with gout. The aim of this study was to carry out an exploration of the footwear experiences of people with gout.

**Methods:**

A qualitative descriptive methodological approach was used for both data collection and analysis. A purposive sampling strategy was adopted with semi-structured interviews conducted, involving 11 participants with gout. Thematic analysis was employed to identify key meanings and patterns within the data.

**Results:**

Four key themes derived from interviews included; (1) comfort as a priority, (2) knowing what to buy, (3) knowing what to wear, and (4) challenges of different environments. Footwear comfort was of great importance and linked to characteristics of footwear, with uncomfortable footwear negatively influencing participation in daily activities. The balancing of comfort, appearance and cost, led to less options and reduced confidence when shoe shopping. Footwear use was further limited by the presence of foot tophi and flares, resulting in compromise of footwear choice. Environments such as formal settings and the workplace, led to different footwear requirements.

**Conclusion:**

People with gout experience problems with footwear which can impact many aspects of life. Health care professionals should consider these footwear-related issues to help facilitate those with gout in finding appropriate footwear.

**Electronic supplementary material:**

The online version of this article (10.1186/s13047-019-0349-7) contains supplementary material, which is available to authorized users.

## Background

Gout is a common form of inflammatory arthritis characterised by deposition of monosodium urate (MSU) crystals, which form in people with high serum urate levels (hyperuricaemia). Gout can present as intermittent episodes of acute arthritis (gout flares) and/or subcutaneous nodules of MSU crystals (tophi) [1]. People with gout experience high levels of foot pain, impairment and disability [2]. To reduce the impact of gout related foot pain, appropriately designed footwear has been used [3]. However, it has been found that people with gout frequently wear footwear which is ill-fitting, lacks cushioning and lacks support. This may be due to inappropriate design at the point of purchase, or the wearing of the footwear over time resulting in effective design components becoming ineffective. Factors related to footwear may contribute to the high levels of foot pain, impairment and disability [4].

Footwear is an important concern for people with gout, with previous qualitative work highlighting footwear-related issues such as the inability to wear footwear during gout flares [5–7], uncertainty about what footwear type and design [8, 9], and difficulty finding footwear which accommodates for foot tophi [5, 9]. Further, in a previous mixed-methods study using an online survey with open-ended questions people with gout reported difficulty finding suitable shoes, revealed the impact of shoes on activity and identified what they preferred in relation to footwear features [10]. Although the impact of gout on footwear choice and use has been described, there is limited understanding of the experiences and perceptions of footwear of people living with gout. The aim of this study was to explore the personal experiences of footwear in people with gout.

## Methods

### Design

This qualitative study sought to gain insight into the subjective experiences of people living with gout in obtaining and wearing footwear [11]. Semi-structured interviews were conducted to explore the participants’ individual perspectives. Inclusion criteria were: gout according to the 1977 preliminary American Rheumatism Association criteria [6], ≥20 years of age, and no history of other inflammatory arthritis or neuromuscular disease. Those who are unable to provide informed consent were excluded. Ethical approval was obtained from the Auckland University of Technology Ethics Committee (14/233) and all participants provided written informed consent.

### Participants

Participants were recruited through public newspaper advertising in Auckland, New Zealand using purposeful sampling. Eligible participants were selected to achieve diversity across the following characteristics; gender, ethnicity, disease duration, presence of foot tophus, serum urate, frequency of gout flares.

### Data collection

Face-to-face interviews were undertaken either at the Auckland University of Technology or their home. Interviews were conducted by MF, who is an experienced podiatrist and has previously been involved in footwear studies of people with gout. The interviews were audio-recorded. Initial discussions with the participant were held to determine a shared definition of footwear and direct them towards the area of interest. Participants were invited to bring pairs of their own footwear to further enhance discussion. Interview questions were developed based on previous studies in gout [3, 4]. An opening question of “… tell me about your experiences of footwear?” was asked, followed by additional trigger questions (open-ended and directed), and the opportunity for participants to express additional ideas they felt were important (Additional file 1). Questions were designed to promote two-way dialogue when exploring areas of interest, with regular summarises of the content discussed shared with the participant during interviews. The interviews lasted between 20 and 90 min.

### Data analysis

Data collection and analysis occurred simultaneously and iteratively, and it emerged that this created new insights and additional dialogue, which influenced subsequent interviews and analyses. Interviews continued until the authors considered that sufficient information power was achieved by the clear research aim, diversity of participants across the sampling framework and the depth of discussions during interviews. Data was analysed using inductive thematic analysis [12] which aligns with qualitative description [13]. Audio recordings of the interviews were transcribed verbatim, anonymised to ensure confidentiality, and analysed after each interview. Transcripts were read and re-read to immerse the researcher in the data. Transcripts were then manually coded by MF, with initial codes and concepts reviewed by a second author (AW). Generated codes were then grouped into potential themes and sub-themes. These were then reviewed to determine a clear distinction between each theme. The final themes were defined, named and agreed upon by all authors. Illustrative quotes from transcripts were selected to provide evidence of each theme.

## Results

**Fig. 1:**
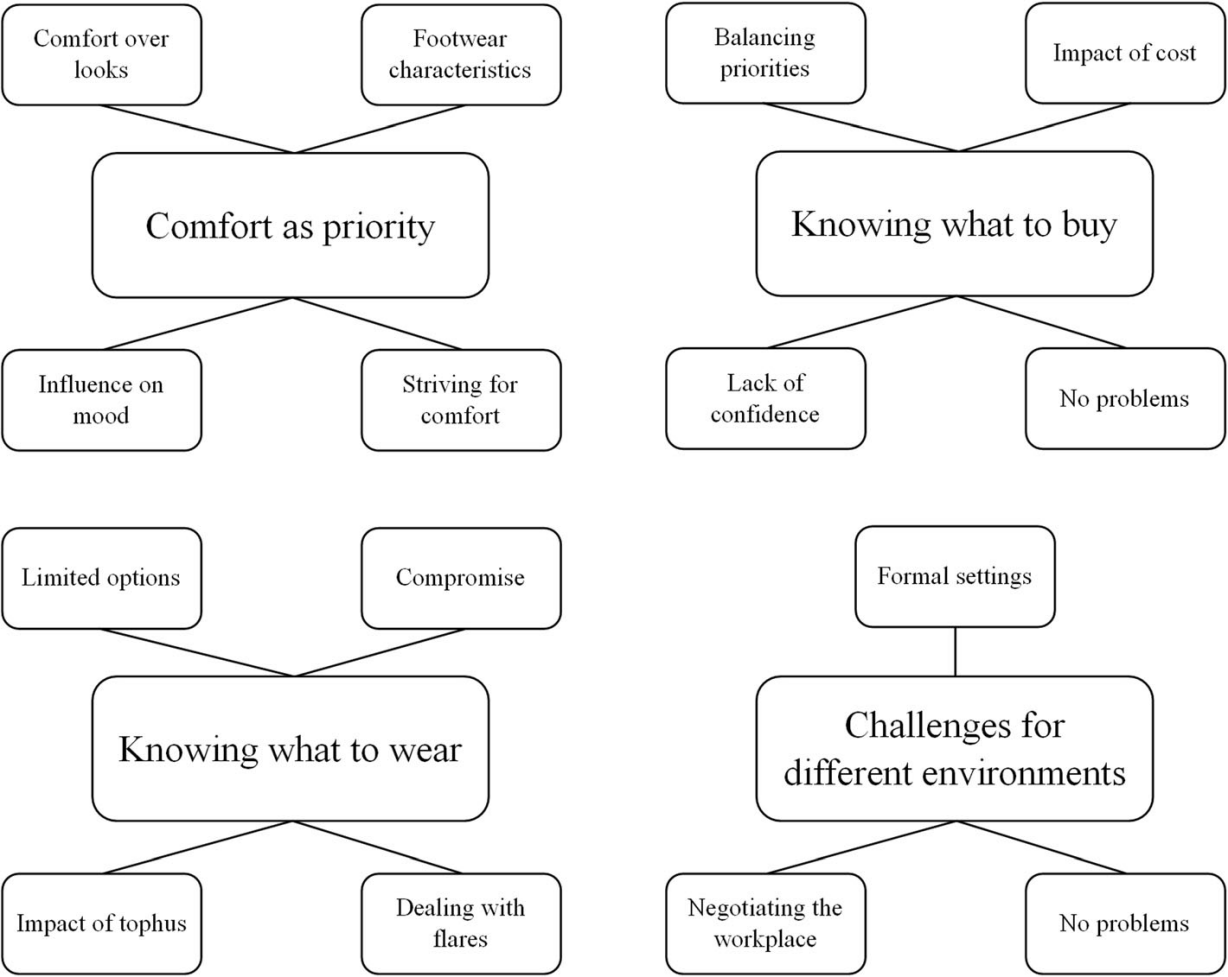
Thematic map showing the central four themes and sub-themes

Nine males and two females were interviewed. There was diversity across age, gender, ethnicity, and clinical features, consistent with the sampling framework (Table 1). Four central themes were derived from the data; (1) comfort as a priority, (2) knowing what to buy, (3) knowing what to wear, and (4) challenges of different environments. The thematic map showing the four central themes and sub-themes is displayed in Fig. 1.

**Table 1:** Participant demographics

Participant	Gender	Age (years)	Ethnicity	Disease duration (years)	Tophus	Serum urate (mmol/L)	Flare frequency (past year)
1	F	61	South African	12	Y	0.27	3
2	M	54	Māori	25	N	0.40	3
3	M	83	Māori	10	N	0.27	2–3
4	M	40	NZ European	3	N	0.43	5
5	M	49	Pacific Island	3	N	0.45	2–3
6	M	40	Pacific Island	10	Y	0.59	6
7	F	53	Māori	2	N	0.41	1–2
8	M	72	NZ European	10	N	0.29	1–2
9	M	58	NZ European	5	N	0.54	0
10	M	48	Pacific Island	20	N	0.36	6
11	M	70	NZ European	15	Y	a	0

^a^serum urate not recently checked

### Comfort as a priority

All participants stated the importance of comfort, supporting the idea that having comfortable footwear was a priority. The concept of striving for comfort was evident, with feelings of satisfaction upon finding comfortable footwear:

*“I didn’t realise that you can have comfortable shoes, cause I’ve never had comfortable shoes before” Participant 10, Male, 48 years old*

For some, feeling comfortable was more important than ‘looking good’:

*“I’ve been wearing shoes in the past that don’t look good, but they are comfortable. That’s, and then, I mean I always get eyes and looks and weirds, but I didn’t really care I was just like ‘oh, I’m comfortable man’” Participant 6, Male, 40 years old*

Footwear characteristics such as having good fit, cushioning, being lightweight and having enough room to accommodate the foot, were identified as important influencers of footwear comfort:

*“The fit of the shoe is important if it’s too, if it’s too tight then it’s not comfortable” Participant 8, male, 72 years old*

Having uncomfortable footwear led to foot pain, which in turn could influence one’s mood and ability to participate. This consequence was viewed with frustration:

*“I'm the person who has to sit with a problem when I get home tonight because my feet are sore, and then I can't sleep, and then you don't sleep, and then you're miserable as H the next morning, and then you've got to work, and you're grumpy” Participant 1, Female, 61 years old*

### Knowing what to buy

Barriers to shoe shopping were described including budgetary constraints, limited range, and a lack of confidence in knowing what the right shoe is to buy. Finding a balance between comfort and appearance was frequently described:

*“The shoe looked really primo, but I knew straight away even with the bigger size fitting it was really uncomfortable. I thought, no I can’t put myself through this cause I’ll end up with very sore feet you know and ah so I didn’t buy them” Participant 10, Male, 48 years old*

For others, this balance was strongly influenced by cost, placing further limitations on the footwear available forcing some to ‘work with’ what was left:

*“I think it’s just my um, my budget wise. What am I able to afford um, compared to what is out of my price range” Participant 6, Male, 40 years old*

Obtaining advice was difficult creating uncertainty surrounding the right type of footwear to buy. This resulted in a lack of confidence with purchases based on negative past experiences, such as footwear becoming uncomfortable shortly after leaving the shop:

*“I can try something on in the store and think ‘oh my god this is so comfortable, fantastic, problem solved’ and then, um it might not be for two or three wears then I’ll be walking, and that pain will come back and it’s like if I don’t take these shoes off well it’s just going to escalate” Participant 7, Female, 53 years old*

In contrast, some found shoe shopping relatively straight-forward, with gout playing little role in the decision-making process when purchasing footwear:

*“I haven’t even really thought about buying shoes related to the gout” Participant 11, Male, 70 years old*

### Knowing what to wear

Despite owning multiple pairs of shoes, participants described a lack of suitable options with respect to the footwear in the cupboard. Having gout meant that footwear which was previously suitable, was no longer appropriate:

*“In terms of shoes pre-gout, the only shoes I can still wear are these, that I had before I ever had gout” Participant 7, Female, 53 years old*

Those with tophi described difficulties in accommodating for deformity, and how affected sites were irritated by certain footwear:

*“If I go out I’ll wear leather, proper leather shoes. Trouble is with that bump on my toe it’s a bit of a pain aye. You know, um, very restrictive actually” Participant 6, Male, 40 years old*

The unpredictable nature of not knowing whether footwear would remain comfortable or exacerbate their foot problems was described. For some, inappropriately fitting footwear could lead to a flare:

*“I wore those, and it basically, again longer shoe but not enough width so that just aggravated it and kind of spoiled a day or two of the holiday, because my foot was flared up” Participant 4, Male, 40 years old*

For some, there was resignation that finding footwear compatible with their foot and beliefs may not be possible, with others accepting that their current footwear may be as good as it gets. Compromise was evident:

*“Having this gout there’s not much, there’s not much around. It’s almost like here’s what you’ve got to try and fit into, try to make it part of you, sort of um your footwear” Participant 6, Male, 40 years old*

### Challenges of different environments

Participants described that their footwear requirements were different depending on the situation. In formal settings, there was tension between having comfortable footwear and maintaining appearance. The trade-off of sacrificing footwear comfort was to put up with the pain during and after the occasion:

*“You’ve got a formal or a fancy event to go to, you kind of, you just sacrifice as I’ve said earlier you deal with the consequence tomorrow because this looks right or this is more appropriate for that activity so you just basically suck it up and consequences come tomorrow” Participant 4, Male, 40 years old*

Health and safety requirements dictated the footwear choices for several participants. Steel cap boots were viewed as limiting due to being heavy, inflexible and restrictive in the forefoot. Some would adapt their footwear habits to accommodate for their gout symptoms during a flare:

*“When I got the gout, I still go to the work, one safety boot, one sneaker” Participant 2, Male, 54 years old*

For others footwear discomfort resulted in a change in workplace practice:

*“Even though I can work with the footwear I don’t stay on my feet as long, so I’ll try and stay on the hoist, I’ve changed my-my work structure to-to suit the ailment” Participant 5, Male, 49 years old*

In contrast, some participants did not report any significant issues as they had found footwear which was comfortable and acceptable for the environments in which they interacted:

*“I don’t ah spend a lot of time thinking about my shoes I wear them and that’s that. And once I, and once you’ve got a comfortable pair you don’t need to think a lot about it” Participant 3, Male, 83 years old*

## Discussion

This study offers unique insights into the footwear experiences and the footwear-related issues of people with gout, with four themes described. Factors contributing towards comfortable and acceptable footwear were readily identified, however, the practicalities of finding and choosing footwear which met these requirements was challenging. There was uncertainty in knowing what the best footwear was and whether footwear would exacerbate foot problems.

Participants placed footwear comfort as a priority, which aligns with previous research [4]. However, what was additionally revealed is that ‘comfort’ was linked to individual footwear characteristics, supporting the concept that particular footwear characteristics help to reduce the burden of foot pain and disability in those with gout [3, 10].

Our findings demonstrate that some people with gout struggle with finding appropriate footwear, aligning with previous research [9, 10]. When shopping, there was a desire to find footwear that met requirements for both comfort and appearance. Factors such as cost [4, 10] added further constraints on footwear choice. Participants described limited footwear options and reduced confidence with their footwear purchases, which may help to explain the high occurrence of poor-quality footwear worn by people with gout [4].

Prior studies have shown the impact of gout flares [6] and tophi [5, 9] on footwear choices. Participants in this study described similar narratives, and we also found that footwear could in turn exacerbate gout symptoms. Having gout meant that participants’ footwear needed to meet their current foot health status rather than their previous footwear expectations.

The impact of footwear extended beyond foot symptoms. There was an inseparable link between the participant’s footwear and their clothing outfit, meaning footwear decisions for social occasions were often made to the detriment of comfort. Where health and safety requirements determined choice, strategies such as not wearing a safety boot during flares demonstrates how footwear can be a limiting factor, resulting in potentially unsafe workplace behaviour. This is a particular challenge in gout, which frequently affects men of working age [14] adding another element to the difficulty that people with gout face when managing their gout symptoms and maintaining employment participation [7, 9].

We found some participants did not have any foot problems or difficulty with footwear and others who did not consider gout in their decision-making surrounding footwear, even if their gout is problematic. This appears to contrast previous studies highlighting the difficulties encountered by people with gout [5–8, 10], however, is similar to other work [9] reporting a diversity of experience with gout, and that not everyone with gout has foot problems or has the same foot problems. This suggests a need for more individualised approaches based on the patient experience.

The lack of suitable options both when purchasing footwear and lack of choice in those already owned was acknowledged by participants. Potential solutions to assist in finding appropriate footwear have been proposed for people with foot problems [15]. Health care practitioners involved in foot health and footwear can use this information to help those with gout reduce the disease burden on foot health. Footwear manufacturers and those in the retail setting should consider the challenges that people with gout face in finding suitable footwear.

Potential limitations of this study are that it was conducted in an urban region and may not represent the experiences of people in rural locations who have different footwear needs. The participants’ occupation and socioeconomic status were not part of the sampling framework, however, we acknowledge that these may have an influence on footwear experiences and issues [15]. Another factor not captured is the possible influence of any previous footwear education on the results. However, it is clear that despite any previous education, these participants still experience difficulties. Hence, given the difficulties experienced by the participants of this study, future work is needed to develop footwear education for people with gout. Participants were aware at the time of recruitment that the study was about footwear experiences, and those with negative experiences may have been more interested in participating, therefore, the study findings may not be generalisable to all people with gout.

## Conclusions

People with gout experience problems with footwear which can impact many aspects of life. Gout can limit a person’s ability to find comfortable footwear which is acceptable and attainable. In addition, the environment in which people interact presents additional challenges to achieving comfortable footwear. Health care professionals should consider these footwear-related issues to help facilitate those with gout in finding appropriate footwear.

## Additional file

Additional file 1: Interview guide. (DOCX 14 kb)

## Data Availability

Data and material available for this study would require further approval upon request from the corresponding author.
