# Supplementary material for: The footwear experiences of people with gout: a qualitative study
Source: J Foot Ankle Res. 2019 Jul 12;12:38. doi: 10.1186/s13047-019-0349-7 (PMC6625048; doi:10.1186/s13047-019-0349-7)
Supplement: Supplementary file 1 — Interview guide. (DOCX 14 kb) [file 13047_2019_349_MOESM1_ESM.docx]

**Additional file 1.** Interview guide

1. Definition of footwear
   1. Discussion to orientate the participant towards the topic of interest
      1. What shoes do you normally wear?
      2. Asking the participant to bring out their shoes (if comfortable doing so)
2. Opening question (with additional prompts/probes)
   1. Tell me about your experiences of footwear
      1. Has this changed post gout diagnosis?
3. Trigger questions (with additional prompts/probes)
   1. What are the most important things you look for in footwear?
   2. What feelings do you have about the footwear currently available to you?
      1. Where do you find your footwear?
   3. What barriers have you experienced related to footwear?
   4. What effect has footwear had on your feet?
   5. What impact has footwear had on your ability to do the things you wanted to do?
      1. Work, daily living, social settings, exercise
   6. Design features of an ‘ideal shoe’
4. Participant driven questions/topics
   1. Any other points that the participant wishes to raise and/or discuss
5. Conclusion of interview (with a summary of main points)
